# The genome sequence of a ground beetle, *Leistus spinibarbis *(Fabricius, 1775)

**DOI:** 10.12688/wellcomeopenres.19997.1

**Published:** 2023-09-19

**Authors:** Maxwell V. L. Barclay, Natural History Museum Genome Acquisition Lab, Darwin Tree of Life Barcoding collective, Wellcome Sanger Institute Tree of Life programme, Wellcome Sanger Institute Scientific Operations: DNA Pipelines collective, Tree of Life Core Informatics collective, Darwin Tree of Life Consortium

**Affiliations:** 1 Natural History Museum, London, England, UK

**Keywords:** Leistus spinibarbis, a ground beetle, genome sequence, chromosomal, Coleoptera

## Abstract

We present a genome assembly from an individual male *Leistus spinibarbis* (a ground beetle; Arthropoda; Insecta; Coleoptera; Carabidae). The genome sequence is 235.1 megabases in span. Most of the assembly is scaffolded into 23 chromosomal pseudomolecules, including the X sex chromosome. The mitochondrial genome has also been assembled and is 15.82 kilobases in length. Gene annotation of this assembly on Ensembl identified 23,576 protein coding genes.

## Species taxonomy

Eukaryota; Metazoa; Eumetazoa; Bilateria; Protostomia; Ecdysozoa; Panarthropoda; Arthropoda; Mandibulata; Pancrustacea; Hexapoda; Insecta; Dicondylia; Pterygota; Neoptera; Endopterygota; Coleoptera; Adephaga; Caraboidea; Carabidae; Nebriinae; Nebriini; *Leistus*; *Leistus spinibarbis* (Fabricius, 1775) (NCBI:txid878056).

## Background

The genus *Leistus* Frölich, 1799 is divided into six subgenera, and includes more than 250 species of medium sized, fast running predatory ground beetles, mainly distributed in the Palaearctic region ( Anichtchenko *et al.*, 2021). *Leistus spinibarbis* is the type species of the subgenus *Pogonophorus* Latreille, 1802, and is itself divided into three subspecies ( Löbl & Löbl, 2017). The British population forms part of the nominate subspecies *Leistus spinibarbis spinibarbis*, which has a restricted distribution in the centre and west of Europe, being listed for only nine countries: Great Britain, Belgium, the Netherlands, Luxembourg, France, Austria, Germany, Monaco and Italy ( Löbl & Löbl, 2017). Other subspecies occur to the south and east of this range. In the British Isles the species is widespread in England and Wales, local in Scotland and apparently absent from Ireland.

Leistus are specialist predators of springtails (Collembola) and have expanded mandibles used for catching this prey, and are hence sometimes called ‘plate-jaws’ (e.g. Telfer & Walters, 2010). These authors use the name ‘Prussian Plate-Jaw’ for *L. spinibarbis*, because of the metallic ‘Prussian blue’ reflection on the black cuticle. It is a conspicuous beetle, on average larger and more brightly coloured than any of the other five species of *Leistus* that occur in Britain.

*Leistus spinibarbis* is 8 to 10.5 mm in length, winged and able to fly, and can be found in woods, gardens and near the coast ( Luff, 2007). In the author’s experience, it can often be found in degraded and human-modified habitats in and around cities, and is usually encountered singly and by chance rather than by deliberate searching. The specimen used for the genome was collected on a wall adjoining a busy London road, surrounded by housing and gardens. The genome of a ground beetle, *Leistus spinibarbis*, was sequenced as part of the Darwin Tree of Life Project, a collaborative effort to sequence all named eukaryotic species in the Atlantic Archipelago of Britain and Ireland. Here we present a chromosomally complete genome sequence for *Leistus spinibarbis*, based on one male specimen from Fulham, London, UK.

## Genome sequence report

**Figure 1.**
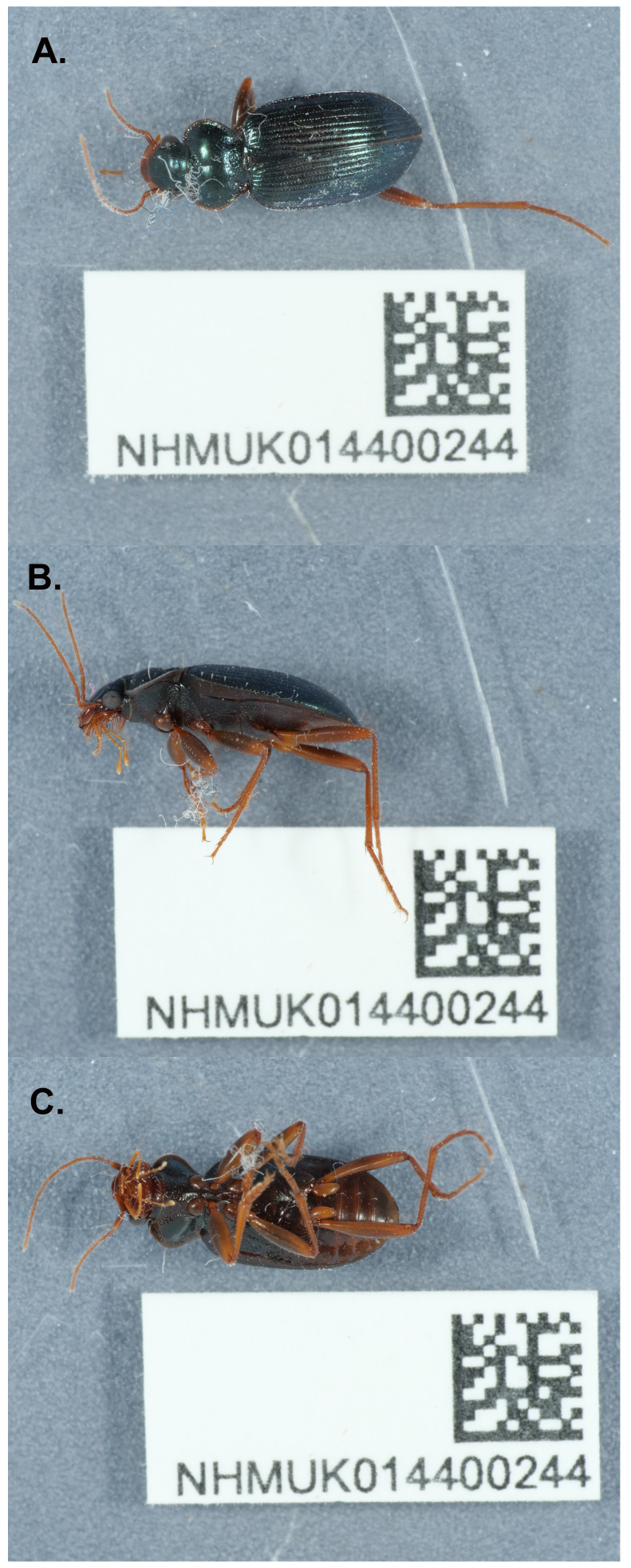
Photographs of the *Leistus spinibarbis* (icLeiSpin1) specimen used for genome sequencing.

The genome was sequenced from one male *Leistus spinibarbis* ( Figure 1) collected from Fulmead Street, Fulham, London, UK (51.48, –0.19). A total of 66-fold coverage in Pacific Biosciences single-molecule HiFi long reads was generated. Primary assembly contigs were scaffolded with chromosome conformation Hi-C data. Manual assembly curation corrected 19 missing joins or mis-joins and removed two haplotypic duplications, reducing the scaffold number by 5.43%, and increasing the scaffold N50 by 16.49%.

**Figure 2.**
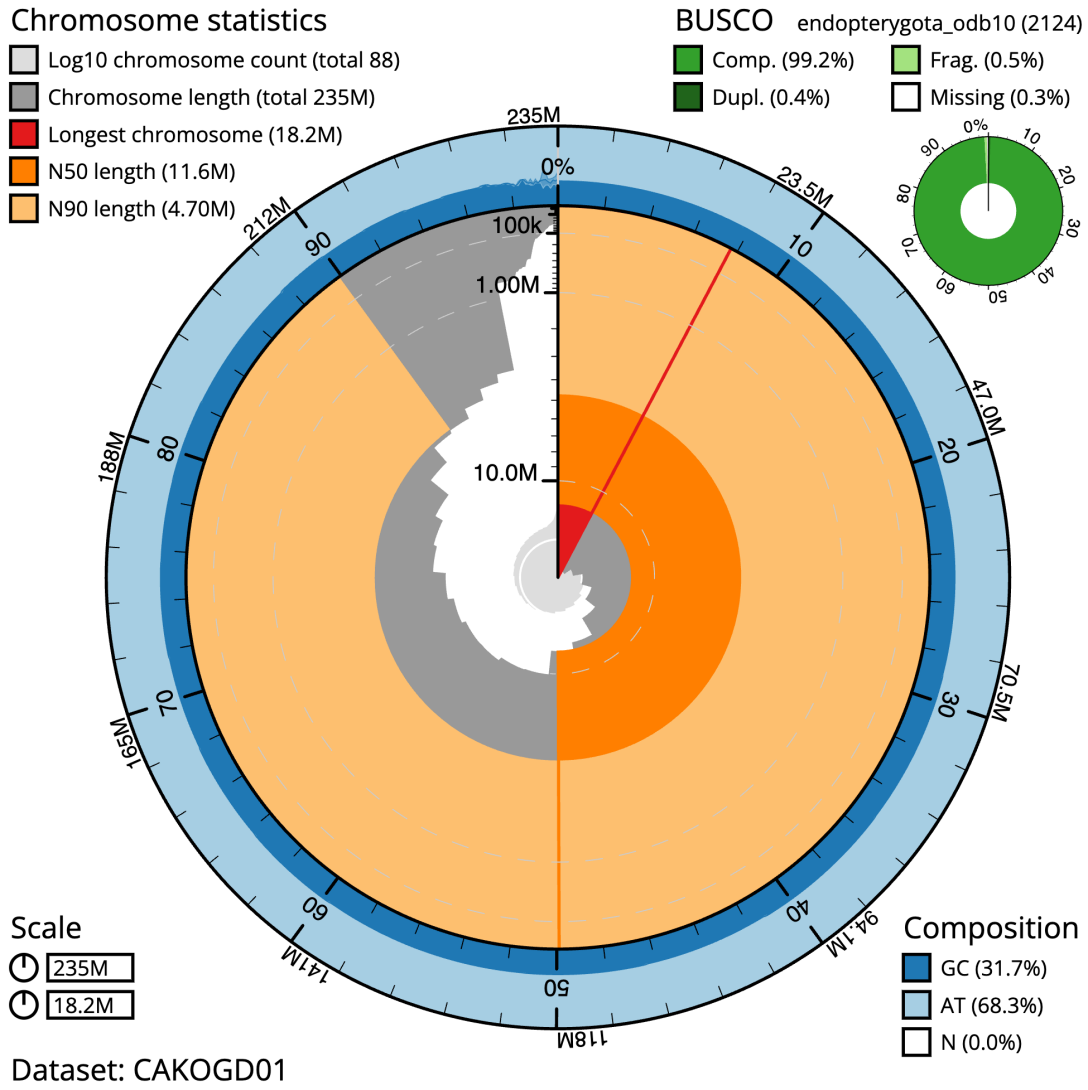
Genome assembly of *Leistus spinibarbis*, icLeiSpin1.1: metrics. The BlobToolKit Snailplot shows N50 metrics and BUSCO gene completeness. The main plot is divided into 1,000 size-ordered bins around the circumference with each bin representing 0.1% of the 235,131,629 bp assembly. The distribution of scaffold lengths is shown in dark grey with the plot radius scaled to the longest scaffold present in the assembly (18,248,107 bp, shown in red). Orange and pale-orange arcs show the N50 and N90 scaffold lengths (11,648,774 and 4,697,515 bp), respectively. The pale grey spiral shows the cumulative scaffold count on a log scale with white scale lines showing successive orders of magnitude. The blue and pale-blue area around the outside of the plot shows the distribution of GC, AT and N percentages in the same bins as the inner plot. A summary of complete, fragmented, duplicated and missing BUSCO genes in the endopterygota_odb10 set is shown in the top right. An interactive version of this figure is available at https://blobtoolkit.genomehubs.org/view/icLeiSpin1.1/dataset/CAKOGD01/snail.

The final assembly has a total length of 235.1 Mb in 87 sequence scaffolds with a scaffold N50 of 11.6 Mb ( Table 1). Most (96.9%) of the assembly sequence was assigned to 23 chromosomal-level scaffolds, representing 22 autosomes and the X sex chromosome. Chromosome-scale scaffolds confirmed by the Hi-C data are named in order of size ( Figure 2– Figure 5; Table 2This appears to be a male assembly denoted by read coverage for Chromosome X being half that of the autosomes. Despite the presence of unassigned repetitive scaffolds, it was not possible to identify a Y chromosome. As there is no way to reliably determine if this sample is XY or XO the assembly has been submitted with a single X allosome assignment. While not fully phased, the assembly deposited is of one haplotype. Contigs corresponding to the second haplotype have also been deposited. The mitochondrial genome was also assembled and can be found as a contig within the multifasta file of the genome submission.

**Table 1.** Genome data for *Leistus spinibarbis*, icLeiSpin1.1.

Project accession data		
Assembly identifier	icLeiSpin1.1	
Species	*Leistus spinibarbis*	
Specimen	icLeiSpin1	
NCBI taxonomy ID	878056	
BioProject	PRJEB50883	
BioSample ID	SAMEA9359424	
Isolate information	icLeiSpin1, male: abdomen (DNA sequencing), head and thorax (Hi-C scaffolding)	
Assembly metrics *		*Benchmark*
Consensus quality (QV)	54.4	*≥ 50*
*k*-mer completeness	99.99%	*≥ 95%*
BUSCO **	C:99.2%[S:98.7%,D:0.4%], F:0.5%,M:0.3%,n:2,124	*C ≥ 95%*
Percentage of assembly mapped to chromosomes	96.9%	*≥ 95%*
Sex chromosomes	X chromosome	*localised homologous pairs*
Organelles	Mitochondrial genome assembled	*complete single alleles*
Raw data accessions		
PacificBiosciences SEQUEL II	ERR8705868	
Hi-C Illumina	ERR8702791	
Genome assembly		
Assembly accession	GCA_933228885.1	
*Accession of alternate* *haplotype*	GCA_933228875.1	
Span (Mb)	235.1	
Number of contigs	116	
Contig N50 length (Mb)	7.5	
Number of scaffolds	87	
Scaffold N50 length (Mb)	11.6	
Longest scaffold (Mb)	18.3	
Genome annotation		
Number of protein-coding genes	23,576	
Number of gene transcripts	23,931	

* Assembly metric benchmarks are adapted from column VGP-2020 of “Table 1: Proposed standards and metrics for defining genome assembly quality” from ( Rhie *et al.*, 2021). ** BUSCO scores based on the endopterygota_odb10 BUSCO set using v5.3.2. C = complete [S = single copy, D = duplicated], F = fragmented, M = missing, n = number of orthologues in comparison. A full set of BUSCO scores is available at https://blobtoolkit.genomehubs.org/view/icLeiSpin1.1/dataset/CAKOGD01/busco.

**Figure 3.**
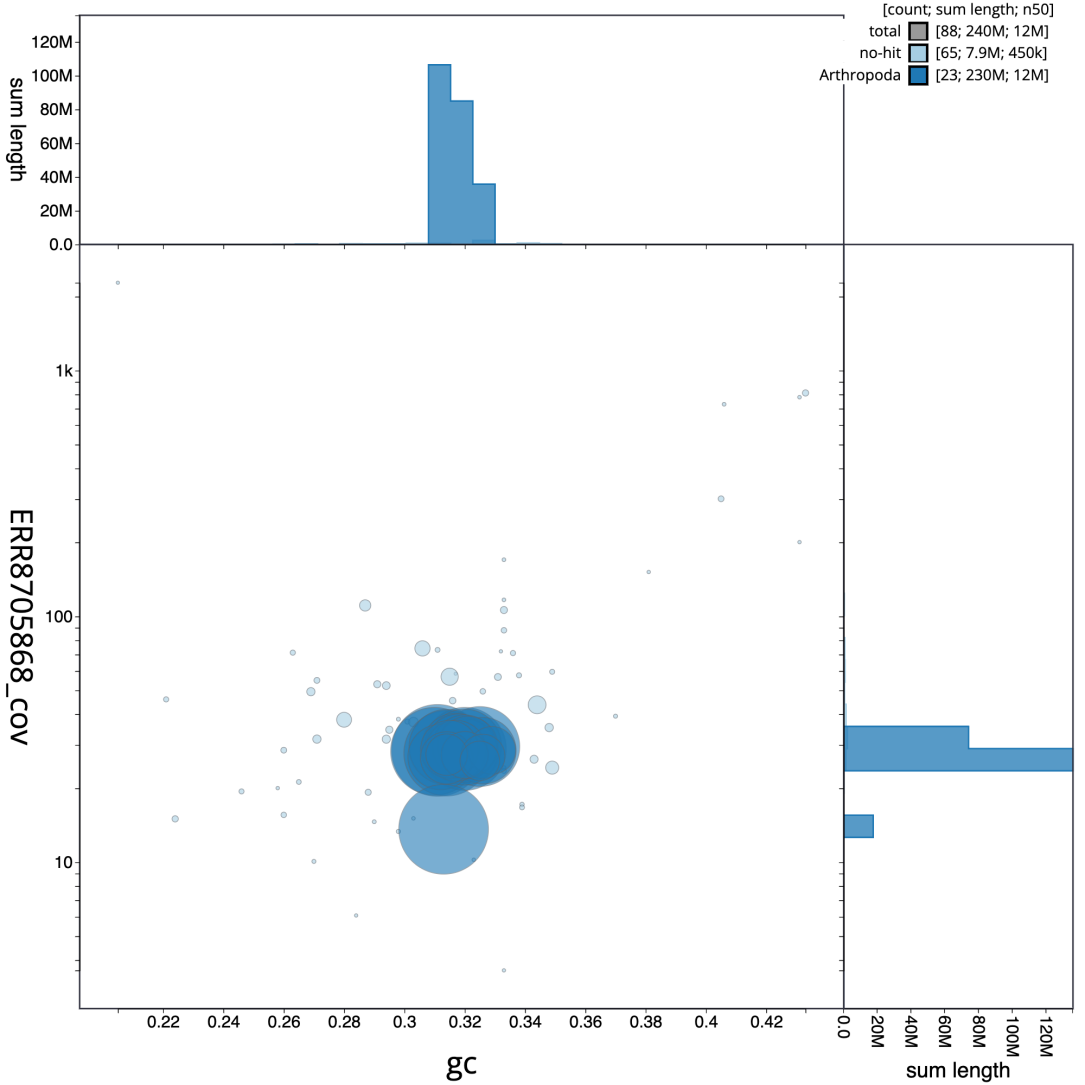
Genome assembly of *Leistus spinibarbis*, icLeiSpin1.1: BlobToolKit GC-coverage plot. Scaffolds are coloured by phylum. Circles are sized in proportion to scaffold length. Histograms show the distribution of scaffold length sum along each axis. An interactive version of this figure is available at https://blobtoolkit.genomehubs.org/view/icLeiSpin1.1/dataset/CAKOGD01/blob.

**Figure 4.**
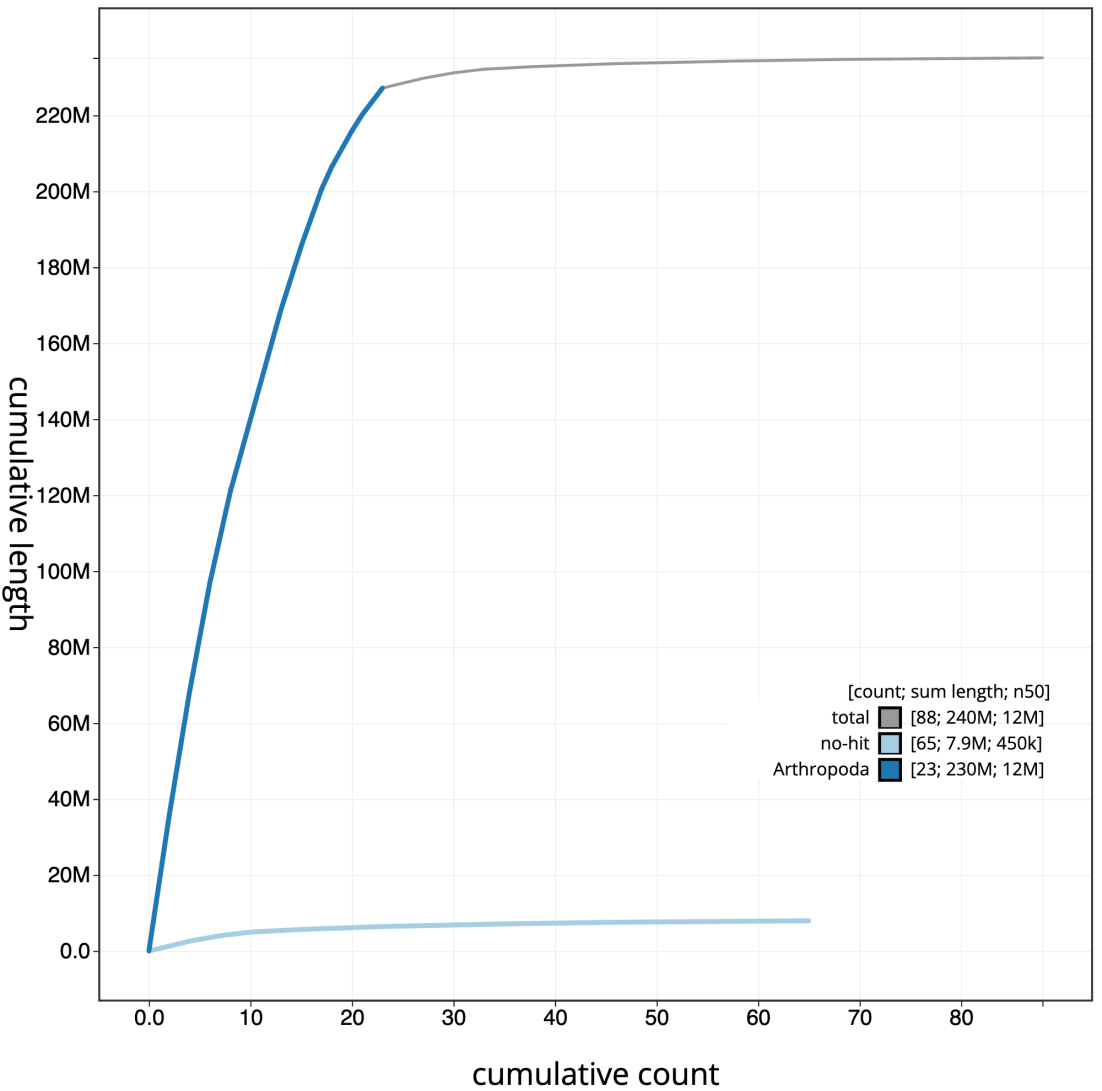
Genome assembly of *Leistus spinibarbis*, icLeiSpin1.1: BlobToolKit cumulative sequence plot. The grey line shows cumulative length for all scaffolds. Coloured lines show cumulative lengths of scaffolds assigned to each phylum using the buscogenes taxrule. An interactive version of this figure is available at https://blobtoolkit.genomehubs.org/view/icLeiSpin1.1/dataset/CAKOGD01/cumulative.

**Figure 5.**
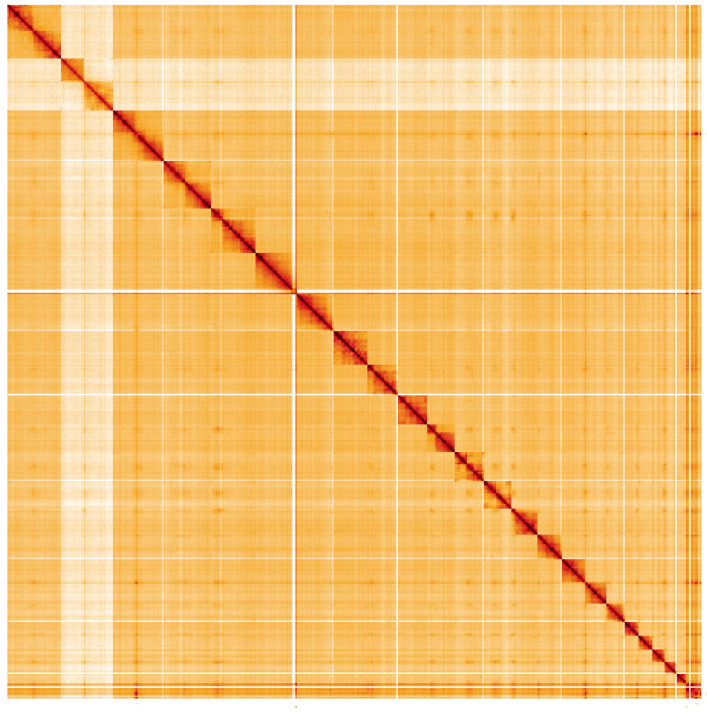
Genome assembly of *Leistus spinibarbis*, icLeiSpin1.1: Hi-C contact map of the icLeiSpin1.1 assembly, visualised using HiGlass. Chromosomes are shown in order of size from left to right and top to bottom. An interactive version of this figure may be viewed at https://genome-note-higlass.tol.sanger.ac.uk/l/?d=FKGrm21hTo6nvptbBrWYKw.

**Table 2.** Chromosomal pseudomolecules in the genome assembly of *Leistus spinibarbis*, icLeiSpin1.

INSDC accession	Chromosome	Length (Mb)	GC%
OW121791.1	1	18.25	31.0
OW121793.1	2	16.71	31.0
OW121794.1	3	15.81	31.5
OW121795.1	4	14.88	32.0
OW121796.1	5	13.8	32.5
OW121797.1	6	12.22	31.5
OW121798.1	7	11.65	32.0
OW121799.1	8	9.86	31.5
OW121800.1	9	9.82	32.0
OW121801.1	10	9.55	32.5
OW121802.1	11	9.53	32.0
OW121803.1	12	9.24	31.0
OW121804.1	13	8.82	32.0
OW121806.1	15	7.98	31.5
OW121805.1	14	7.76	32.0
OW121807.1	16	7.21	31.5
OW121808.1	17	5.87	31.5
OW121809.1	18	4.85	33.0
OW121810.1	19	4.7	32.0
OW121811.1	20	4.2	32.5
OW121812.1	21	3.69	31.5
OW121813.1	22	3.33	32.5
OW121792.1	X	17.44	31.5
OW121814.1	MT	0.02	20.5

The estimated Quality Value (QV) of the final assembly is 54.4 with *k*-mer completeness of 99.99%, and the assembly has a BUSCO v5.3.2 completeness of 99.2% (single =98.7%, duplicated = 0.4%), using the endopterygota_odb10 reference set ( *n* = 2,124).

Metadata for specimens, spectral estimates, sequencing runs, contaminants and pre-curation assembly statistics can be found at https://links.tol.sanger.ac.uk/species/878056.

## Genome annotation report

The *Leistus spinibarbis* genome assembly (GCA_933228885.1) was annotated using the Ensembl rapid annotation pipeline ( Table 1; https://rapid.ensembl.org/Leistus_spinibarbis_GCA_933228885.1/Info/Index). The resulting annotation includes 23,931 transcribed mRNAs from 23,576 protein-coding genes.

## Methods

### Sample acquisition and nucleic acid extraction

A male *Leistus spinibarbis* (specimen ID NHMUK014400244, individual icLeiSpin1) was collected from Fulham, London, UK (latitude 51.48, longitude –0.19) on 2021-05-12. The specimen was collected and identified by Maxwell Barclay (Natural History Museum) and preserved by dry-freezing at –80°C.

DNA was extracted at the Tree of Life laboratory, Wellcome Sanger Institute (WSI). The icLeiSpin1 sample was weighed and dissected on dry ice with tissue set aside for Hi-C sequencing. Abdomen tissue was cryogenically disrupted to a fine powder using a Covaris cryoPREP Automated Dry Pulveriser, receiving multiple impacts. High molecular weight (HMW) DNA was extracted using the Qiagen MagAttract HMW DNA extraction kit. HMW DNA was sheared into an average fragment size of 12–20 kb in a Megaruptor 3 system with speed setting 30. Sheared DNA was purified by solid-phase reversible immobilisation using AMPure PB beads with a 1.8X ratio of beads to sample to remove the shorter fragments and concentrate the DNA sample. The concentration of the sheared and purified DNA was assessed using a Nanodrop spectrophotometer and Qubit Fluorometer and Qubit dsDNA High Sensitivity Assay kit. Fragment size distribution was evaluated by running the sample on the FemtoPulse system.

### Sequencing

Pacific Biosciences HiFi circular consensus DNA sequencing libraries were constructed according to the manufacturers’ instructions. DNA sequencing was performed by the Scientific Operations core at the WSI on a Pacific Biosciences SEQUEL II (HiFi) instrument. Hi-C data were also generated from head and thorax tissue of icLeiSpin1 using the Arima2 kit and sequenced on the Illumina NovaSeq 6000 instrument.

### Genome assembly, curation and evaluation

Assembly was carried out with Hifiasm ( Cheng *et al.*, 2021) and haplotypic duplication was identified and removed with purge_dups ( Guan *et al.*, 2020). The assembly was then scaffolded with Hi-C data ( Rao *et al.*, 2014) using YaHS ( Zhou *et al.*, 2023). The assembly was checked for contamination and corrected as described previously ( Howe *et al.*, 2021). Manual curation was performed using HiGlass ( Kerpedjiev *et al.*, 2018) and Pretext ( Harry, 2022). The mitochondrial genome was assembled using MitoHiFi ( Uliano-Silva *et al.*, 2023), which runs MitoFinder ( Allio *et al.*, 2020) or MITOS ( Bernt *et al.*, 2013) and uses these annotations to select the final mitochondrial contig and to ensure the general quality of the sequence.

A Hi-C map for the final assembly was produced using bwa-mem2 ( Vasimuddin *et al.*, 2019) in the Cooler file format ( Abdennur & Mirny, 2020). To assess the assembly metrics, the *k*-mer completeness and QV consensus quality values were calculated in Merqury ( Rhie *et al.*, 2020). This work was done using Nextflow ( Di Tommaso *et al.*, 2017) DSL2 pipelines “sanger-tol/readmapping” ( Surana *et al.*, 2023a) and “sanger-tol/genomenote” ( Surana *et al.*, 2023b). The genome was analysed within the BlobToolKit environment ( Challis *et al.*, 2020) and BUSCO scores ( Manni *et al.*, 2021; Simão *et al.*, 2015) were calculated.

Table 3 contains a list of relevant software tool versions and sources.

**Table 3.** Software tools: versions and sources.

Software tool	Version	Source
BlobToolKit	3.4.0	https://github.com/ blobtoolkit/blobtoolkit
BUSCO	5.3.2	https://gitlab.com/ezlab/ busco
Hifiasm	0.16.1-r375	https://github.com/ chhylp123/hifiasm
HiGlass	1.11.6	https://github.com/higlass/ higlass
Merqury	MerquryFK	https://github.com/ thegenemyers/MERQURY.FK
MitoHiFi	2	https://github.com/ marcelauliano/MitoHiFi
PretextView	0.2	https://github.com/wtsi- hpag/PretextView
purge_dups	1.2.3	https://github.com/dfguan/ purge_dups
sanger-tol/ genomenote	v1.0	https://github.com/sanger- tol/genomenote
sanger-tol/ readmapping	1.1.0	https://github.com/sanger- tol/readmapping/tree/1.1.0
YaHS	yahs-1.1.91eebc2	https://github.com/c-zhou/ yahs

### Genome annotation

The BRAKER2 pipeline ( Brůna *et al.*, 2021) was used in the default protein mode to generate annotation for the *Leistus spinibarbis* assembly (GCA_933228885.1) in Ensembl Rapid Release.

### Wellcome Sanger Institute – Legal and Governance

The materials that have contributed to this genome note have been supplied by a Darwin Tree of Life Partner. The submission of materials by a Darwin Tree of Life Partner is subject to the **‘Darwin Tree of Life Project Sampling Code of Practice’**, which can be found in full on the Darwin Tree of Life website here. By agreeing with and signing up to the Sampling Code of Practice, the Darwin Tree of Life Partner agrees they will meet the legal and ethical requirements and standards set out within this document in respect of all samples acquired for, and supplied to, the Darwin Tree of Life Project. 

Further, the Wellcome Sanger Institute employs a process whereby due diligence is carried out proportionate to the nature of the materials themselves, and the circumstances under which they have been/are to be collected and provided for use. The purpose of this is to address and mitigate any potential legal and/or ethical implications of receipt and use of the materials as part of the research project, and to ensure that in doing so we align with best practice wherever possible. The overarching areas of consideration are:

• Ethical review of provenance and sourcing of the material

• Legality of collection, transfer and use (national and international) 

Each transfer of samples is further undertaken according to a Research Collaboration Agreement or Material Transfer Agreement entered into by the Darwin Tree of Life Partner, Genome Research Limited (operating as the Wellcome Sanger Institute), and in some circumstances other Darwin Tree of Life collaborators.

## Data Availability

European Nucleotide Archive: *Leistus spinibarbis*. Accession number PRJEB50883; https://identifiers.org/ena.embl/PRJEB50883. ( Wellcome Sanger Institute, 2022) The genome sequence is released openly for reuse. The *Leistus spinibarbis* genome sequencing initiative is part of the Darwin Tree of Life (DToL) project. All raw sequence data and the assembly have been deposited in INSDC databases. Raw data and assembly accession identifiers are reported in Table 1.

## Author information

Members of the Natural History Museum Genome Acquisition Lab are listed here: https://doi.org/10.5281/zenodo.4790042.

Members of the Darwin Tree of Life Barcoding collective are listed here: https://doi.org/10.5281/zenodo.4893703.

Members of the Wellcome Sanger Institute Tree of Life programme are listed here: https://doi.org/10.5281/zenodo.4783585.

Members of Wellcome Sanger Institute Scientific Operations: DNA Pipelines collective are listed here: https://doi.org/10.5281/zenodo.4790455.

Members of the Tree of Life Core Informatics collective are listed here: https://doi.org/10.5281/zenodo.5013541.

Members of the Darwin Tree of Life Consortium are listed here: https://doi.org/10.5281/zenodo.4783558.
