# Supplementary material for: The genome sequence of a ground beetle, Leistus spinibarbis (Fabricius, 1775)
Source: Wellcome Open Res. 2023 Sep 19;8:412. [Version 1] doi: 10.12688/wellcomeopenres.19997.1 (PMC10594052; doi:10.12688/wellcomeopenres.19997.1)
Supplement: Peer review history

# Reviewer report: Reviewer response for version 1

**DOI:** 10.21956/wellcomeopenres.22143.r69002

**Published:** 2023-11-13

**Authors:** Arun Sethuraman, Trevor Mugoya

**Affiliations:** 1 Department of Biology, San Diego State University, San Diego, California, USA; 2 Biology, San Diego State University, San Diego, California, USA

This is a well written manuscript that describes a high-quality genome of *Leistus spinibarbis*, with ample details on sample collection, library preparation, sequencing, assembly, QC, and annotation. I commend the authors on this nice work, and hope that this will be a very useful contribution to researchers studying Coleopterans. The data was all accessible, which was very helpful.

 The one comment that we had was the unavailability of code for their computational pipelines (for assembly, annotation, QC, and other steps). I recommend that the authors make this publicly available via GitHub or some similar platform, especially to ensure reproducibility/reusability of their data.

Are sufficient details of methods and materials provided to allow replication by others?

Partly

Is the rationale for creating the dataset(s) clearly described?

Yes

Are the datasets clearly presented in a useable and accessible format?

Yes

Are the protocols appropriate and is the work technically sound?

Yes

Reviewer Expertise:

Genetics, genomics, computational biology

We confirm that we have read this submission and believe that we have an appropriate level of expertise to confirm that it is of an acceptable scientific standard.

# Reviewer report: Reviewer response for version 1

**DOI:** 10.21956/wellcomeopenres.22143.r68998

**Published:** 2023-10-23

**Authors:** Danilo T Amaral

**Affiliations:** 1 Departamento de Biologia, Centro de Ciências Humanas e Biológicas, Universidade Federal de Sao Carlos, São Carlos, State of São Paulo, Brazil

I had the pleasure of reviewing the manuscript by Barclay *et al.,* titled "The genome sequence of a ground beetle, *Leistus spinibarbis* (Fabricius, 1775)." This study presents the a new chromosome-level genome assembly for a member of the Carabidae family, offering valuable insights into the genome structure of this group. The high-quality assembly of genomes, particularly at the chromosome level, is pivotal in advancing scientific research. Mainly with the chromosome X definition and mitochondrial genome characterization accurately deciphering the complete genetic blueprint of an organism enables researchers to gain a comprehensive understanding of its structure, function, and evolutionary history. The manuscript provided well-written and detailed methodologies and results.

 As a reader, I have a minor suggestion. While the figures and tables effectively demonstrate the assembly quality, it may be beneficial to include Figure 4 as supplementary material, based on the sequencing and assembly methodologies. Additionally, it would be interesting to know if any previous studies have performed the karyotyping of this species (or closely related species) to determine the number of chromosomes. Including this information in the text would further enhance the study's context and significance.

Are sufficient details of methods and materials provided to allow replication by others?

Yes

Is the rationale for creating the dataset(s) clearly described?

Yes

Are the datasets clearly presented in a useable and accessible format?

Yes

Are the protocols appropriate and is the work technically sound?

Yes

Reviewer Expertise:

Genomic and bioinformatic

I confirm that I have read this submission and believe that I have an appropriate level of expertise to confirm that it is of an acceptable scientific standard.

# Reviewer report: Reviewer response for version 1

**DOI:** 10.21956/wellcomeopenres.22143.r67439

**Published:** 2023-09-26

**Authors:** Sean Schoville

**Affiliations:** 1 Department of Entomology, University of Wisconsin-Madison, Madison, Wisconsin, USA

This is a nice resource for beetle (especially Carabid beetle) phylogenomics research, and the assembly is of high quality.

 Now that several genomes are available for *Nebria*, I wonder if an analysis of synteny across *Nebria *and *Leistus *could be added to this analysis. The lack of a clear Y chromosome is interesting, so my thoughts were based on if that might yield insights to the XO vs XY hypothesis (one would expect synteny at all autosomes in your dataset).

**Suggestions on Background:**

 As part of your justification, you might consider the important phylogenetic placement of Leistus within the diverse tribe Nebriini:

 Kavanaugh, D.H., Maddison, D.R., Simison, W.B., Schoville, S.D., Schmidt, J., Faille, A., Moore, W., Pflug, J.M., Archambeault, S.L., Hoang, T. and Chen, J.Y., 2021. Phylogeny of the supertribe Nebriitae (Coleoptera, Carabidae) based on analyses of DNA sequence data. *ZooKeys*, *1044*, p.41.

 Its also an example of convergence, as it is one of three lineages who demoinstrate specialized feeding on Collembola:

 Baulechner, D., Jauker, F., Neubauer, T.A. and Wolters, V., 2020. Convergent evolution of specialized generalists: implications for phylogenetic and functional diversity of carabid feeding groups. *Ecology and Evolution*, *10*(20), pp.11100-11110.

 My favorite descriptor that I have heard/read is that Leistus has a "Collembola basket". The prey-capture technique has been described well by:

 Bauer, Thomas. “Beetles which use a setal trap to hunt springtails: The hunting strategy and apparatus of Leistus (Coleoptera, Carabidae).” *Pedobiologia* (1985): n. pag.

 Leistus is often found in cool, damp and rock habitats. Interestingly, it seems to prefer foraging on sloped and elevated features when foraging, such as vegetation, on large boulders or sloped banks, similar to your rock wall.

 Buse, J., Daume, N., Eckerter, T., Friesdorf, C., Fritze, M.A., Höfer, H., Kastner, L., Kaus-Thiel, A., Ludewig, H.H., Popa, F., Schlör, A. & Dreiser, C. (2020): **New records of *Leistus montanus* Stephens, 1827 (Coleoptera: Carabidae) in Germany and a first insight into its habitat preference at local and landscape scale**. Angewandte Carabidologie 13: 67-74

**Suggestions Genome Sequence Report:**

 Typographical error: Insert period and space between: Table 2This

**Suggestions Genome Annotation Report:**

 I'd suggest clarifying that annotations are based solely on protein homology, not RNAseq data.

Are sufficient details of methods and materials provided to allow replication by others?

Yes

Is the rationale for creating the dataset(s) clearly described?

Partly

Are the datasets clearly presented in a useable and accessible format?

Yes

Are the protocols appropriate and is the work technically sound?

Yes

Reviewer Expertise:

Evolutionary genomics of insects, including Carabid beetles.

I confirm that I have read this submission and believe that I have an appropriate level of expertise to confirm that it is of an acceptable scientific standard.

## References

[1] : Phylogeny of the supertribe Nebriitae (Coleoptera, Carabidae) based on analyses of DNA sequence data. *Zookeys* .2021;1044: 10.3897/zookeys.1044.62245 41-152 10.3897/zookeys.1044.62245 34183875PMC8222211

[2] : Convergent evolution of specialized generalists: Implications for phylogenetic and functional diversity of carabid feeding groups. *Ecol Evol* .2020;10(20) : 10.1002/ece3.6746 11100-11110 10.1002/ece3.6746 33144951PMC7593144

[3] : Beetles which use a setal trap to hunt springtails: The hunting strategy and apparatus of Leistus (Coleoptera, Carabidae). *Pedobiologia* .1985;28(4) : 10.1016/S0031-4056(23)06550-2 275-287 10.1016/S0031-4056(23)06550-2

[4] : New records of Leistus montanus Stephens, 1827 (Coleoptera: Carabidae) in Germany and a first insight into its habitat preference at local and landscape scale. *Angewandte Carabidologie* .2020;67-74
